# Urinary extracellular vesicles carry valuable hints through mRNA for the understanding of endocrine hypertension

**DOI:** 10.3389/fendo.2023.1155011

**Published:** 2023-03-29

**Authors:** Simonetta Friso, Annalisa Castagna, Gabriele Mango, Oliviero Olivieri, Francesca Pizzolo

**Affiliations:** Department of Medicine, University of Verona, Verona, Italy

**Keywords:** 11-beta hydroxysteroid dehydrogenase 2, HSD11B2, endocrine hypertension, gene expression, mRNA, exosomes

## Abstract

Urinary extracellular vesicles (uEVs), released from cells of the urogenital tract organs, carry precious information about originating tissues. The study of molecules transported through uEVs such as proteins, lipids and nucleic acids provides a deeper understanding of the function of the kidney, an organ involved in the pathogenesis of hypertension and a target of hypertension-mediated organ damage. Molecules derived from uEVs are often proposed for the study of disease pathophysiology or as possible disease diagnostic and prognostic biomarkers. Analysis of mRNA loading within uEVs may be a unique and readily obtainable way to assess gene expression patterns of renal cells, otherwise achievable only by an invasive biopsy procedure. Interestingly, the only few studies investigating transcriptomics of hypertension-related genes through the analysis of mRNA from uEVs are inherent to mineralocorticoid hypertension. More specifically, it has been observed that perturbation in human endocrine signalling through mineralcorticoid receptors (MR) activation parallels changes of mRNA transcripts in urine supernatant. Furthermore, an increased copy number of uEVs-extracted mRNA transcripts of the 11β-hydroxysteroid dehydrogenase type 2 (HSD11B2) gene were detected among subjects affected by apparent mineralocorticoid excess (AME), a hypertension-inducing autosomal recessive disorder due to a defective enzyme function. Moreover, by studying uEVs mRNA, it was observed that the renal sodium chloride cotransporter (NCC) gene expression is modulated under different conditions related to hypertension. Following this perspective, we illustrate here the state of the art and the possible future of uEVs transcriptomics towards a deeper knowledge of hypertension pathophysiology and ultimately more tailored investigational, diagnostic-prognostic approaches.

## 1 Introduction

Urinary extracellular vesicles (uEV) are a mixture of membrane-bound lipid secretory vesicles that are released by the cells of the urogenital tract and then excreted in the urine (1). In addition to the information carried through lipids and proteins expressed on their membranes, uEVs function also as envelopes that carry valuable information from the tissue of origin by transporting specific characterizing cellular proteins and nucleic acids, in particular RNA, either mRNAs or miRNAs into the urine (2). Based on differences in biogenesis, composition, size and functions, the extracellular vesicles are classified into three families: microvesicles, exosomes and apoptotic bodies.

As for the extracellular vesicles detected in urine (uEVs), they have been extensively studied in recent years particularly those belonging to the exosome family, because they represent a valuable source of data on urine secretory cells and ultimately on the physiology and pathophysiology of the kidney and the genitourinary system. Urinary EVs have been studied primarily as carriers of molecules that may serve as biomarkers, all of which are potentially helpful for diagnostic purposes in kidney diseases such as: acute kidney injury, renal fibrosis, diabetic nephropathy, but also for the rarest IgA nephropathy, congenital kidney diseases, lupus nephritis, focal segmental glomerulosclerosis, and neoplastic diseases of the urinary system such as those affecting prostate, bladder and kidney.

The kidney is also a crucial organ for the study of such an important common multiorgan disease as hypertension. Indeed, kidney tissue is both a target for the hypertension-mediated organ damage, and a major player in water and sodium balance, the latter mechanism classically compromised in the context of mineralocorticoid hypertension.

Therefore, the possibility of exploring the uEVs cargo to investigate the physiology and pathophysiology of hypertension is of particular value, mostly considering that such information is obtained through a readily available and non-invasive procedure such as the collection of a urine sample. Thus, uEVs are a precious source for kidney tissue analysis, as they can be considered as a liquid biopsy (3).

Indeed nucleic acids, and specifically RNA, are more stable when carried within uEVs than those detected from cells freely isolated from urine, probably due to a protective function from degradation exerted by the lipid bilayer on vesicular cargo.

Therefore, uEVs can be a unique and easily obtainable way to assess gene expression analysis of a tissue so strictly related to the pathophysiology of arterial hypertension.

A number of reports have identified mRNA transcripts specific to each portion of the nephron in uEVs confirming the function of uEVs as carriers of gene transcriptional information and consequently of specific disease biomarkers, potentially useful for diagnostic purposes of diseases affecting the urinary system (4–7).

In this perspective, starting from the results available in the literature so far, we propose to consider the high relevance of information derived from uEVs mRNA analysis in order to highlight and propose possible novel approaches to investigate arterial hypertension and more specifically endocrine hypertension.

## 2 Approaching endocrine hypertension by the analysis of mRNA from uEVs

Despite the growing number of studies focusing their interest on the molecular mechanisms underlying arterial hypertension through the analysis of proteins or nucleic acid products obtained from uEVs, only few studies have focused on the expression of hypertension-related genes through the analysis of mRNA obtained from uEVs. Interestingly, however, all those studies are inherent to mineralocorticoid hypertension. A summary of the findings available from the literature on uEVs mRNAs in endocrine hypertension is reported in **Table 1**.

**Table 1:** Summary of the reports on uEVs mRNAs in endocrine hypertension.

Gene	Protein	Function	Effects on pathophysiology of mineralocorticoid hypertension	Reference
HSD11B2	11-beta-hydroxysteroid dehydrogenase type 2	It converts active steroids (i.e., cortisol and corticosterone) into their inactive metabolites, increasing the MR selectivity for aldosterone and thus regulating epithelial sodium transport	↑ AME	(8)
SCNN1A	Sodium channel epithelial 1 subunit alpha	It encodes for the α subunit of ENaC	↓ Prehypertensive patients after sodium loading	(3)
SCNN1G	Sodium channel epithelial 1 subunit gamma	It encodes for the γ subunit of ENaC	↓ Prehypertensive patients after sodium loading	(3)
SGK1	Serum/Glucocorticoid Regulated Kinase 1	It increases renal Na^+^ retention and K^+^ elimination by regulating ion channels	↓ Prehypertensive patients after sodium loading	(3)
SLC12A3	Na^+^-Cl^−^ Cotransporter	It is involved in the reabsorption of Na^+^ and Cl^-^ at kidney level	↑ Healthy control vs hypertensive patients	(9)
			↑ APA after adrenalectomy	
			↓ Hypertensive patients after sodium loading	
TSC22D3	TSC22 domain family protein 3	It modulates ENaC activity	↓ Prehypertensive patients after sodium loading	(3)

MR, mineralocorticoid receptor; AME, Apparent Mineralocorticoid Excess; ENaC, Epithelial sodium Channel; APA, Aldosterone Producing Adenoma. ↑, upregulation; ↓, downregulation.

### 2.1 Mineralcorticoid receptors stimulation in pre-hypertension

In a study of pre-hypertensive subjects, the change in mineralocorticoid receptors (MR) stimulation achieved by following a low- or a high-sodium diet, was paralleled by activation or inhibition of the renin angiotensin aldosterone system (RAAS) and was accompanied by changes in transcriptional signatures in mRNA derived from uEVs. More specifically, a low sodium intake resulted in an increase of the RAAS activation mediated through aldosterone signalling *via* MR, whereas a sodium load inhibited the system, with concomitant modulation of the expression of a set of MR-controlled genes. The Authors suggested that these findings imply the identification of non-invasive putative biomarkers of the altered renal and cardiorenal physiology (3). Some caution is definitely needed before transferring these findings to clinical practice, partly because RNA isolation was performed on urine supernatant without a specific step to isolate uEVs. However, the approach of using transcriptomic data with mRNA from uEVs certainly highlights crucial perspectives for the study of mineralocorticoid receptors modulation in hypertension.

### 2.2 HSD11B2 gene expression in *apparent mineralocorticoid excess* disease

A rare form of hypertension due to an autosomal recessive inherited disorder, namely the apparent mineralocorticoid excess (AME) syndrome, is caused by an impaired function of the enzyme 11β-hydroxysteroid dehydrogenase type 2 (11β-HSD2) (10). The activity of 11β-HSD2 enzyme is traditionally assessed by measuring either the urinary cortisol metabolites ratio (tetrahydrocortisol + allotetrahydrocortisol/tetrahydrocortisone, THF+5aTHF/THE) or the urinary cortisol/cortisone (F/E) ratio (10, 11). The defective enzyme activity that characterizes the disease is defined by low renin hypertension due to an excessive MR activation by cortisol. In recent years a milder form of the disease due to partial 11β-HSD2 deficiency was also described, which has been termed nonclassical AME (NCAME) and in which analysis of uEVs loading has revealed a possible role of several noncoding RNAs (12–14).

We subsequently described, for the first time, the isolation and expression of HSD11B2 gene by detecting mRNA isolated from uEVS in members of an AME family characterized by the 662C>G HSD11B2 mutation (15) involving a change from alanine to glycine at position 221 in the amino acid sequence. HSD11B2 mRNA expression of uEVs has been shown to be closely correlated with the different genotypes so that the two homozygous mutant probands showed the highest transcriptional levels of uEVs HSD11B2 mRNA. The amount of the HSD11B2 mRNA transcripts isolated from uEVs progressively decreased among heterozygous 221AG hypertensive subjects compared with normotensive heterozygous 221AG, and then 221AA wild-type subjects. Heterozygous hypertensive subjects had higher concentrations of HSD11B2 mRNA transcripts than heterozygous normotensive subjects, suggesting a modulation of exosomal HSD11B2 mRNA associated with both mutation carrier status and hypertensive status. Interestingly, the F/E urinary ratio also correlated with the HSD11B2 mRNA copy number, thus validating the use of the F/E ratio as a possible useful surrogate marker of 11β-HSD2 enzyme activity (8).

### 2.3 The renal sodium chloride cotransporter expression modulation in different sodium balance conditions

The renal sodium chloride cotransporter (NCC, thiazide-sensitive Na/Cl cotransporter), mainly expressed in the distal convoluted tubules (DCT) of the nephron, is a crucial channel for the regulation of sodium retention. Studies on NCC protein suggested that the abundance of NCC in uEVs reflects the state of NCC expression in the renal tubular cells in humans (16–18). In a seminal study, although conducted on a small number of patients, uEVs NCC protein was shown to be higher in patients with primary aldosteronism (PA), as compared with those with essential hypertension, therefore providing support to hypothesize a role for NCC as a marker of aldosteronism (16). In hypertensive patients, modulation of the RAAS by high- or low- sodium diet was able to change, the exosomal content of NCC in parallel (19). Moreover, exogenous mineralocorticoid administration by fludrocortisone was associated with a rapid increase in abundance of NCC and phosphorylated NCC (pNCC), possibly related to the modulation of potassium, that was inversely related to NCC and pNCC (17). Very recently, pNCC and NCC were demonstrated to be more abundant in PA patients with unilateral disease at the adrenal venous sampling than in patients with the bilateral disease, suggesting a possible use of uEVs NCC protein as a biomarker for PA subtypes (20).

Last year, our research group demonstrated in samples of PA patients that the detection of NCC mRNA in uEVs was possible (9). The study showed the modulation of NCC expression in different conditions, i.e., before and after saline infusion, anti-aldosterone pharmacological treatment, and adrenal surgery. In contrast to the initial working hypothesis, no significant differences were found between essential hypertensive (EH) and PA patients. Similarly, NCC mRNA levels did not differ between the PA subtypes but both saline infusion and adrenalectomy lead to an increase in NCC mRNA abundance (9).

## 3 Discussion

Besides lipids and proteins, uEVs also carry RNA molecules and are secreted from renal epithelial cells into the urine, thus providing access to information about kidney tissue physiology and possibly to kidney-related diseases pathophysiology (21, 22). Most studies, however, have focused, on the proteins and non-coding RNA profiles of uEVs and almost all are related to endocrine hypertension referring more specifically to primary aldosteronism (PA) and to apparent mineralocorticoid excess (AME) (12–14, 16, 17, 19, 20, 23, 24).

Interestingly, in those studies that investigated a specific mRNA of uEVs in endocrine hypertension, namely NCC in PA and 11β-HSD2 in AME (9, 15), the results were somewhat different from those expected when looking at the specific protein studies. Previous data on uEVs NCC protein demonstrated that patients with PA are characterized by a higher level of NCC and pNCC in comparison to essential hypertensive patients, with a modulation by both mineralocorticoid administration and potassium levels, and a higher abundance in patients with documented monolateral versus bilateral PA disease (16, 17, 20, 23). Consistent with these findings, one could expect a reflection of the differences in protein abundance in the abundance of the corresponding mRNA, but this did not occur. No differences were documented in the mRNA expression of NCC uEV of patients with PA and EH, nor were differences observed between monolateral and bilateral disease (9). By contrast, a higher NCC mRNA expression was documented in normotensive subjects, suggesting a possible downregulation of NCC transcription in patients with PA, characterized by a volume expansion. This hypothesis is in line with the reduction in NCC mRNA documented after acute sodium expansion by intravenous sodium loading (9). It is possible to speculate that the documented higher abundance in NCC protein in PA is an effect of different modulation of translation and transcription. It is, furthermore, possible that unrecognized different characteristics of the populations object of study, particularly in relation to potassium levels, contributed to differences among studies on uEVs NCC protein and mRNA.

Similarly, when analysing the uEVs 11β-HSD2 mRNA content in an AME family carrying the 662C>G mutation, one would expect to document a reduced mRNA expression in homozygous as compared to heterozygous and to wild-types, in accordance with the severity of the disease. The results for HSD11B2 mRNA transcripts, on the other hand, were surprising, showing an opposite trend than expected based on genotypes. In fact, highest HSD11B2 gene expression (as measured by mRNA transcripts) was observed in 662C>G homozygous, with a decreasing trend for heterozygous and then for wild types (8).

It is possible to hypothesize that the highest levels of HSD11B2 gene expression in patients with the more severe disease mirror an enhanced transcription of the mutated gene, in order to compensate for the impaired enzymatic activity. This interpretation is in line with a finding previously described in the same family affected by AME and with similar observation for another gene (25) where a transcriptional regulation of the gene by the epigenetic methylation at promoter site was confirmed (26) and observed also according to genotype (15, 25), with a lower HSD11B2 promoter methylation in the two homozygous subjects compared to that observed in the wild-type individuals, thus linking genomic and epigenomic marks to phenotypic expression of disease (15, 27). Considering that the gene promoter methylation is usually associated with transcriptional repression, the results are consistent with the higher abundance of HSD11B2 exosomal mRNA observed in the two homozygous probands.

Taken all together, these data allow to speculate that the study of uEVs mRNA in mineralocorticoid hypertension is complementary, rather than confirmative, to the study of uEVs proteins, adding interesting information about the pathophysiology of the hypertensive disease.

Despite the high interest on uEVs transcriptome, most reports on uEVs rely almost exclusively on noncoding RNA, namely miRNA (14), with only few studies focusing on the analysis of mRNA transcripts (6, 28). This is due, at least in part, to the complexity of obtaining mRNA that may be adequate in terms of integrity and quantity for transcriptomic studies, since mRNA is usually present in very exiguous amount in uEVs in comparison to more abundant molecules (i.e., proteins and noncoding RNAs).

Indeed, studies on transcriptomics from uEVs in humans had, at first, the major aim of defining whether uEVs mRNA does parallel the renal mRNA transcripts so that it may ultimately reflect the intracellular renal physiology. In this regard, the study by Bazzel et al., by using for comparison publicly available RNA-Seq data of gene expression in human renal cortex, observed that uEVs mRNA encodes information concordant with the transcriptional activity within the kidney tissue, thus supporting the idea of using uEVs mRNA analysis to identify renal disease-related gene expression profiles (3).

The transcriptomic data obtained from the analysis of uEVs mRNA are, for this reason, particularly attractive to explore the kidney tissue function including the aspects related to hypertension disease physiology and pathophysiology and, eventually, to identify putative biomarkers of altered renal function.

RNA is, in fact, detectable in urine either within and outside cells, although in this latter case RNA is a relatively unstable and easily targetable for degradation by ribonucleases. When carried by uEVs through the urinary tract, the RNA cargo is, instead, well preserved, because of the protection offered by the lipid bilayer structure of the vesicles. This protected mode of RNA transport from the kidney to the urine, therefore, offers the possibility of obtaining transcriptomic analysis otherwise available only through an invasive procedure such as renal tissue biopsy (29).

A crucial topic in the investigation of uEVs concerns the most appropriate methods to measure and detect uEVs concentration and normalization for the analysis of uEVs content, including that of mRNA.

Normalization has always been a topic of the debate in the field of uEVs research, as the concentration of vesicles in urine involves both intrinsic and extrinsic sources of variability. On one hand, a crucial element of intrinsic variability is related with the rate of urine production, which may be extremely variable according to either the physiological water and salt homeostasis or the presence of pathological conditions of dysregulation. On the other hand, extrinsic variability is inherently related to the different uEVs processing protocols, including not only the urine handling and storage, but also the specific methodology chosen for vesicle isolation and characterization.

For these reasons, it is difficult to obtain a consistent way of normalization, and it should be tailored according to the experimental conditions and the phenomenon under investigation.

Several strategies for normalizing quantitative RNA data have been proposed, though it should be kept in mind that the choice of the reference molecule to normalize data could introduce by itself a confounding factor. The debate on what is the best approach to normalize uEVs mRNA is still open (30).

General strategies for uEVs normalization were presented in the latest position paper by the International Society of Extracellular Vesicles (ISEV) (1) and in a recent work by Blijdorp and colleagues (31). Clear guidelines specifically concerning the study of RNA from uEVs are, however, lacking so far. RNA analysis generally relies on three different approaches: RNA-Seq, RNA array and RT-qPCR. The first two are high-throughput methods for which several normalization strategies are available (32). Moreover, the addition of spike-in RNA has been proven to be effective for the normalization of sequencing reads coming from low input material, which is often the case when dealing with uEVs (33). Special attention should be paid to all the steps of the experimental workflow as the different passages can greatly affect the subsequent RNA analysis. In this regard, some reports on uEVs optimization provide valuable information for uEVs isolation to conduct high-throughput downstream RNA analysis (34). On the other hand specific studies for qPCR analysis on uEVs mRNA are yet limited (35).

According to the last ISEV position paper (36), the mechanisms responsible for the RNAs distribution among uEVs and their relative loading are still unknown as well as the extent of the association between EV-RNA content and the RNA profile of the parent cells. Several molecular players are likely involved in the incorporation of RNA into EVs, such as RNA-binding proteins (RBP) and proteins implicated in EVs biogenesis.

In reason of the relative complexity of uEVs RNA research, it seems that uEVs RNA investigation may not to be addressed with an intent of discovering novel biomarkers but rather to obtain information on disease pathophysiology.

**Figure 1:**
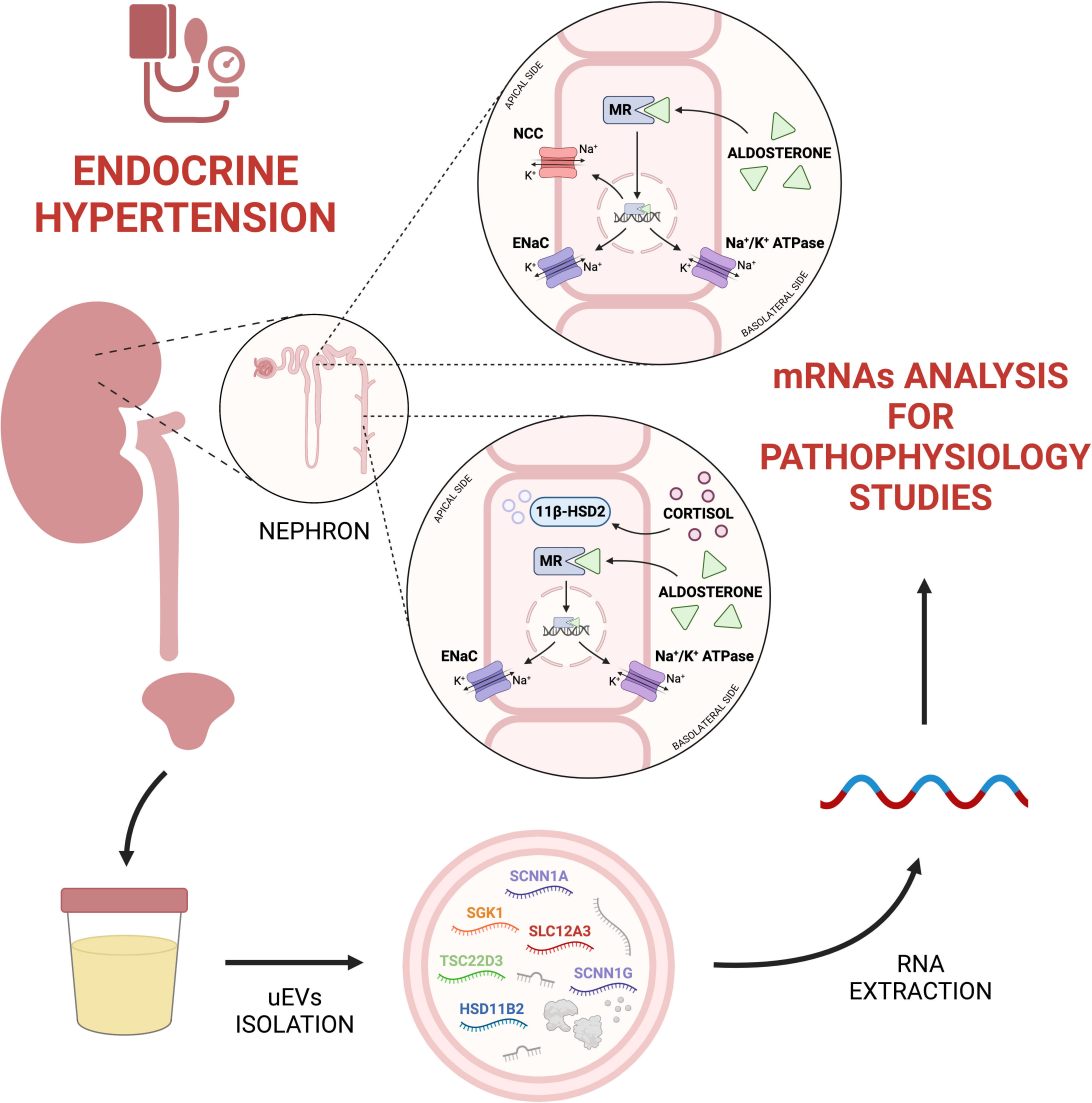
From urine to uEVs transcriptomics in endocrine hypertension. UEVs mRNA analysis can be useful to understand physiopathological processes related to endocrine hypertension. Created with BioRender.com. 11β-HSD2, 11-beta-hydroxysteroid Dehydrogenase type 2; ENaC, Epithelial Sodium Channel; HSD11B2, Hydroxysteroid 11-beta Dehydrogenase 2; MR, Mineralocorticoid Receptor; NCC, Na^+^-Cl^−^ Cotransporter; SCNN1A, Sodium Channel Epithelial 1 Subunit Alpha; SCNN1G, Sodium Channel Epithelial 1 Subunit Gamma; SGK1, Serum/Glucocorticoid Regulated Kinase 1; SLC12A3, Solute Carrier Family 12 Member 3; TSC22D3, TSC22 Domain Family Member 3.

As summarized in **Figure 1**, uEVs are a valuable source of information about the main players involved in mineralocorticoid hypertension, i.e. ion channels and enzymes implied in sodium-water reabsorption along the kidney tubular segments. In reason of the methodological complexity to properly isolate and analyse exosomal mRNA, in our view the utility of uEVs transcriptomic is fundamental to reach a deeper knowledge on the physiology and pathophysiology of mineralocorticoid hypertension than eventually would lead to design more specific diagnostic and prognostic purposes. To date, uEVs RNA research was addressed mainly for the transcription of single genes or predefined groups of genes of interest. A challenge for the next future could be a possible RNA-sequencing approach to explore the entire uEVs transcriptome in patients with mineralocorticoid hypertension, to reveal still unrecognized transcripts of interest.

## Data availability statement

The original contributions presented in the study are included in the article. Further inquiries can be directed to the corresponding author.

## Author contributions

Conceptualization, drafting and critical revision (SF, AC, OO, and FP), drafting, figure drawing and revision of the manuscript (GM). All authors contributed to the article and approved the submitted version.
